# Integrated pest management in western flower thrips: past, present and future

**DOI:** 10.1002/ps.4531

**Published:** 2017-02-28

**Authors:** Sanae Mouden, Kryss Facun Sarmiento, Peter GL Klinkhamer, Kirsten A Leiss

**Affiliations:** ^1^ Research Group Plant Ecology and PhytochemistryInstitute of Biology, Leiden UniversityThe Netherlands

**Keywords:** thrips, Frankliniella occidentalis, integrated pest management, biological control, resistance, ‘omics’ techniques

## Abstract

Western flower thrips (WFT) is one of the most economically important pest insects of many crops worldwide. Recent EU legislation has caused a dramatic shift in pest management strategies, pushing for tactics that are less reliable on chemicals. The development of alternative strategies is therefore an issue of increasing urgency. This paper reviews the main control tactics in integrated pest management (IPM) of WFT, with the focus on biological control and host plant resistance as areas of major progress. Knowledge gaps are identified and innovative approaches emphasised, highlighting the advances in ‘omics’ technologies. Successful programmes are most likely generated when preventive and therapeutic strategies with mutually beneficial, cost‐effective and environmentally sound foundations are incorporated. © 2017 The Authors. *Pest Management Science* published by John Wiley & Sons Ltd on behalf of Society of Chemical Industry.

## 1 INTRODUCTION

Western flower thrips (WFT), *Frankliniella occidentalis* (Pergande), forms a key agri‐ and horticultural pest worldwide. This cosmopolitan and polyphagous invader is abundant in many field and greenhouse crops. WFT developed into one of the most economically important pests owing to its vast damage potential and concurrent lack of viable management alternatives to the pesticide‐dominated methods.^1^ Direct damage results from feeding and oviposition on plant leaves, flowers and fruits, while indirect damage is caused by virus transmission, of which tomato spotted wilt virus (TSWV) is economically the most important.^2^, ^3^ Their small size, affinity for enclosed spaces, high reproductive potential and high dispersal capability cause a high pest pressure.^4^ Control of WFT mainly relied on frequent use of insecticides. This overuse of pesticides has led to the development of WFT resistance to major insecticide groups, residue problems on marketable crops, toxicity towards beneficial non‐target organisms and contamination of the environment.^5^, ^6^, ^7^ Therefore, in the framework of integrated pest management (IPM) programmes, multiple complementary tactics are necessary, including monitoring, cultural, physical and mechanical measures, host plant resistance, biological control and semiochemicals, along with the judicious use of pesticides. IPM programmes for control of WFT have started to develop mainly for protected crops. However, continued injudicious use of pesticides resulted in a resurgence of WFT and associated viruses while depleting its natural enemies and competitive species. As Morse and Hoddle reviewed 10 years ago,^1^ this led to a worldwide destabilisation of IPM programmes for many crops. To emphasise the development and implementation of alternative control measures, the EU issued new legislation on sustainable use of pesticides (Directive 2009/128/EC), as well as on regulation of plant protection products (EC No. 1107/2009). Ten years after Morse and Hoddle, we aim to review the current knowledge about WFT control in relation to IPM, stressing biological control and host plant resistance as areas of major progress. Resulting knowledge gaps are identified, and new innovative approaches, with emphasis on the emerging ‘omics’ techniques, are discussed. WFT biology and ecology, fundamental to the development of knowledge‐based IPM approaches, have already been extensively reviewed elsewhere.^1^, ^4^, ^7^

## 2 WFT CONTROL TACTICS

### 2.1 Monitoring

In order effectively to manage current and anticipate future pest outbreaks, early intervention and the development of economic thresholds are critical. However, the assessment of the economic impact of WFT has only recently begun to develop. Therefore, only a few economic damage thresholds for WFT have been established, such as in tomato, pepper, eggplant, cucumber and strawberry.^8^, ^9^ However, in high‐value ornamental crops or in crops with a high threat of virus transmission, a near‐zero tolerance for WFT prevails.^6^ Monitoring information on the development of WFT population levels relative to the economic thresholds is assessed to decide on the employment of control tactics.^7^ Monitoring is based on regular visual scouting of WFT adults on flowers and fruits or on the use of sticky traps.^10^ Compared with yellow sticky traps, blue traps have shown to catch more WFT; yellow sticky traps can also be used for monitoring aphids, whiteflies and leafminers. The use of monitoring tools has been expanded by the addition of semiochemicals as lures that significantly increase thrips catches.^11^ Based on WFT samplings, models for predictions of WFT population growth and spread of TSWV have been developed as potential decision tools for IPM programmes.^12^

### 2.2 Cultural, mechanical and physical control of WFT

Since ancient time, farmers have been relying on cultural or physical practices for the management of pests. Sanitary practices such as removing weeds, old plant material and debris form the first line of WFT defence.^13^, ^14^ Screening greenhouse openings prevented WFT immigration into protected crops but requires optimisation of ventilation.^15^ WFT incidence in protected tomato was reduced by 20% using greenhouse window screens.^16^ A combination of a positive‐pressure force ventilation system with insect‐proof screens did not prevent greenhouse invasion by thrips.^17^ UV‐reflective mulch repelled WFT colonising adults through interruption of orientation and host‐finding behaviour.^18^, ^19^ Irrigation, creating a less favourable environment for thrips, reduced numbers of WFT adults.^20^ In contrast, high relative humidity favoured WFT larval development and stimulated pupation in the plant canopy.^21^ Fertilisation increases plant development and growth but also affects WFT abundance. Increased levels of nitrogen fertilisation increased WFT population numbers in ornamentals.^22^ Similarly, high levels of aromatic amino acids promoted WFT larval development in different vegetables.^23^ A positive correlation between phenylalanine and female WFT abundance was observed in one study on field‐grown tomatoes, but not in another.^18^, ^24^ High rates of phosphorus favoured thrips development but did not lead to increased thrips damage.^25^ Trap crops draw WFT away from the crop, where it can be controlled more easily.^26^ Flowering chrysanthemums as trap plants lowered WFT damage in a vegetative chrysanthemum crop.^27^ Intercropping French beans with sunflower, potato or baby corn compromised bean yield but reduced damage to the bean pods, increasing marketable yield.^28^

### 2.3 Host plant resistance

Plants and insects have coexisted for more than 350 million years. In the course of evolution, plants have evolved a variety of defence mechanisms, constitutive and inducible, to reduce insect attack, and this has led to host plant resistance. The study of host plant resistance involves a large web of complex interactions, mediated by morphological and chemical traits that influence the amount of damage caused by pests. Understanding the nature of plant defensive traits plays a critical role in designing crop varieties with enhanced protection against pests.

#### 2.3.1 Morphological defence structures

The surface of a host plant can serve as a physical barrier through morphological traits such as waxy cuticles and/or epidermal structures including trichomes. WFT damage was negatively correlated with the amount of epicuticular wax on gladiolus leaves.^29^ Induction of type VI glandular trichomes in response to methyljasmonate application trapped higher numbers of WFT.^30^ However, other studies did not observe any correlation between WFT feeding damage and morphological traits such as hairiness, leaf age, dry weight and leaf area.^31^, ^32^ Instead, the latter provided clear indications that resistance was mainly influenced by chemical host plant composition.

#### 2.3.2 Chemical host plant resistance

Plant chemical defence can arise from both primary and secondary metabolites. Primary metabolites, as nutritional chemicals, are generally beneficial for thrips. However, at low concentrations they can also be involved in WFT resistance. Among different crops, low concentrations of aromatic amino acids were correlated with reduced WFT feeding damage.^23^ Nevertheless, these universal compounds do not provide any uniqueness and are not likely to be effective in resistance on their own. Therefore, the majority of studies focus on the role of secondary metabolites in plant defence. Hitherto, few studies have investigated chemical host plant resistance to WFT. In a study on different chrysanthemum varieties, isobutylamide was suggested to be associated with WFT host plant resistance.^33^ Developing an ecometabolomic approach comparing metabolomic profiles of resistant and susceptible plants, compounds for constitutive WFT resistance were identified and validated in subsequent *in vitro* bioassays.^34^ Identified compounds included jacobine, jaconine and kaempferol glucoside in the wild plant species *Jacobaea vulgaris*, chlorogenic‐ and feroluylquinic acid in chrysanthemum, acyl sugars in tomato and sinapic acid, luteolin and *β*‐alanine in carrot.^31^, ^33^, ^35^, ^36^ Interestingly, some of these metabolites not only have shown a negative effect on WFT but also have received considerable attention for their antioxidant functions in human health prevention.

#### 2.3.3 Transgenic plants

Plant protease inhibitors (PIs) are naturally occurring plant defence compounds reducing the availability of amino acids for insect growth and development. Transgenic alfalfa, expressing an anti‐elastase protease inhibitor, noticeably delayed WFT damage.^37^ Purified cystatin and equistatin, when incorporated into artificial diets, reduced WFT oviposition rates.^38^ Transgenic chrysanthemums, overexpressing multicystatin, a potato proteinase inhibitor, did not show a clear effect on WFT fecundity.^39^ Cysteine PI transgenic potato plants overexpressing stefin A or equistatin were deterrent to thrips, while overexpression of kininogen domain 3 and cystatin C did not inhibit WFT.^40^ Expression of multidomain protease inhibitors in potato significantly improved resistance to thrips.^41^ However, the potential interference of these multidomain proteins with basic cell functions has hindered a practical application for pest management so far. Targeting virus resistance, transgenic tomato expressing G_N_ glycoprotein interfered with TSWV acquisition and transmission by WFT larvae.^42^ The use of transgenic plants, alternated or simultaneously used with additional strategies, is recognised as a promising approach for thrips and tospovirus management by the scientific community. However, highly restrictive political and regulatory frameworks limit the commercialisation of genetically modified crops in Europe.

#### 2.3.4 Induced resistance

In addition to constitutive defences, plants use inducible defences as a response to pest attack, presumably to minimise costs. Induced defences are regulated by a network of cross‐communicating signalling pathways. The plant hormones salicylic acid (SA) and jasmonic acid (JA), as well as ethylene (ET), trigger naturally occurring chemical responses protecting plants from insects and pathogens. The JA pathway plays an important role in defence against thrips. The JA‐responsive genes *VSP2* and *PDF1.2* were strongly stimulated upon exposure of *Arabidopsis* plants to thrips.^43^ WFT reached maximal reproductive performance in the tomato mutant *def‐1*, deficient in JA, in comparison with the mutant expressing a 35S::prosystemin transgene, constitutively activating JA defence.^44^ In contrast to WFT, TSWV infection in *Arabidopsis* induced SA‐regulated gene expression.^43^ The resulting antagonistic interaction between the JA‐ and SA‐regulated defence systems in response to TSWV infection enhanced the performance of WFT preferring TSWV‐infected plants over uninfected ones.^45^ Treatments with exogenous elicitors activate the natural defensive response of a plant, thereby enhancing resistance to thrips. Application of JA in tomato resulted in a decreased preference, performance and abundance of WFT.^46^ Treatment of tomato with acibenzolar‐*S*‐methyl (ASM), a functional analogue of SA, reduced TSWV incidence, but did not influence WFT population densities.^47^ Induced resistance has recently gained more interest and might be of particular value in conjunction with other IPM approaches.

### 2.4 Biological control

Biological control uses the augmentative release of natural enemies as well as conservation approaches to sustain their abundance and efficiency. A large number of natural enemies are known to attack WFT, which can be separated into two groups: macrobials, which include predators and parasitoids, and microbials, which are subdivided into enthomopathogenic fungi and nematodes. Table 1 summarises the most common commercially available biocontrol agents used against WFT.

**Table 1:** Biological control agents of F. occidentalis. Information retrieved from the Biopesticide Database of the University of Hertfordshire (www.herts.ac.uk)

		Classification	Type of agent	WFT stage affected	First use	Commercially available
Predators	Crop dwellers	Mites (foliar)	*Amblyseius cucumeris*	First‐instar larvae	1995	Worldwide
			*Amblyseius barkeri*	First‐instar larvae	1981	Worldwide
			*Amblyseius degenerans*	Larvae	1993	Worldwide
			*Amblyseius californicus*	Larvae	1985	Europe
			*Amblyseius swirskii*	First‐ and second‐instar larvae	2005	Europe
			*Amblyseius andersoni*	Larvae	2007	The Netherlands
			*Amblyseius montdorensis*	Larvae	2010–2011	The Netherlands
			*Amblydromalus limonicus*	Larvae	2010–2011	The Netherlands
		Minute bugs	*Orius insidious*	Larvae and adults	1900s	North America
			*Orius laevigatus*	Larvae and adults	1900s	Worldwide
			*Orius albidipennis*	Larvae and adults	1991	Europe
			*Orius majusculus*	Larvae and adults	1993	EU and USA
			*Orius armatus*	Larvae and adults	2008–2009	Australia
	Soil dwellers	Mites	*Macrocheles robustulus*	Pupae	2008	Europe
			*Hypoaspis aculeifer*	Pupae	1995	Europe
			*Hypoaspis miles*	Pupae	1994	Europe
		Rove beetle	*Atheta coriaria*	Pupae	2002	Canada
Parasitoids		Parasitic wasp	*Ceranisus menes*	Parasitises larvae	1996	The Netherlands
			*Ceranisus americensis*	Parasitises larvae	1996	The Netherlands
Entomopathogens		Nematodes	*Steinernema feltiae*	Pupae, prepupae and larvae	2005	Worldwide
		Fungi	*Lecanicillium lecanii*	Adults most susceptible	2012	Europe
			*Metarhizium anisopliae*	Adults most susceptible	2012	The Netherlands
			*Beauveria bassiana*	Adults most susceptible	2012	EU and USA
			*Isaria fumosorosea*	Larvae	2012	The Netherlands

#### 2.4.1 Predatory mites

The principal arthropod predators associated with WFT biological control are phytoseiid mites (*Amblyseius* spp.) and pirate bugs (*Orius* spp.). Several species of *Amblyseius* have been recorded as predators of WFT, and various species have been assessed for their efficacy. The first predatory mites used for WFT control were *Amblyseius barkeri* and *Neoseiulus* (formerly *Amblyseius) cucumeris*, which primarily feed upon first‐instar larvae. Owing to the inadequate control achieved, a number of other mites have been studied in order to find a superior WFT predator. Species such as *A. limonicus*, *A. swirskii*, *A. degenerans* and *A. montdorensis* proved to be effective predators of WFT.^48^, ^49^ Compared with *N*. *cucumeris*, *A. swirskii* proved to be a better WFT predator than in sweet pepper, as females showed a higher propensity to attack and kill WFT larvae.^50^ In chrysanthemum, *A. swirskii* provided higher thrips control than *N. cucumeris* in summer, likely owing to a better survival, while both predators showed similar efficacy in winter.^51^ Efficiency of *A. swirskii* as a WFT biocontrol agent is also influenced by host plant species, and here increased trichome densities hinder mite performance.^52^ Thrips can also consume *A. swirskii* eggs, and female predators were observed preferentially to oviposit at sites without thrips, or to kill more thrips at oviposition sites, presumably to protect their offspring.^53^ Thrips are not the best food source for mites. Therefore, the addition of supplemental food to *A. swirskii* has recently been investigated. Supplying pollen improved the performance of *A. swirskii* in control of WFT in chrysanthemum, as did the addition of decapsulated brine shrimp cysts (*Artemia* sp.).^54^ Next to being an efficient predator of WFT, *A. swirskii* is easily reared, which allows economic mass production.^49^ Since its commercial introduction in 2005, *A. swirskii* has therefore become the main predator used for biological control of WFT in vegetables and ornamentals worldwide.^49^ In addition to control of WFT, *A. swirskii* also provides control of whiteflies. Although the presence of whitefly can lead to a short‐term escape of thrips from predation, thrips control is not negatively affected by the presence of whitefly, while in contrast *A. swirskii* is a better predator on whitefly in the presence of thrips.^55^, ^56^

#### 2.4.2 Predatory bugs

*Orius*, commonly known as pirate bugs, are known to be generalist predators, preying on adults and larvae of a wide range of insect species such as aphids, whiteflies, spider mites and thrips. Several species of *Orius* have been tested to evaluate their use against WFT. Observations from field and glasshouse experiments in sweet pepper demonstrated that *O. insidious* suppressed WFT almost to extinction, but failed to control WFT properly under short‐day conditions in autumn as they enter diapause.^57^ In contrast, *O. laevigatus* has been successful in all‐year‐round biological control of WFT in vegetables and ornamentals.^58^, ^59^ Success of *Orius* in ornamentals depends on the complexity of flower structure.^59^ Oviposition of *O. laevigatus* has been shown to induce WFT resistance in tomato through wound response.^60^ Although a key natural enemy in biocontrol of WFT, *Orius* spp. are relatively expensive to mass rear.^59^

#### 2.4.3 Soil‐dwelling predators

Most research on WFT biocontrol has focused on adult and larval stages. However, WFT spend one‐third of their life as pupae in the soil. Different soil‐dwelling predatory mites have been investigated, of which *Macrocheles robustulus*, *Stratiolaelaps scimitus* (formerly *Hypoaspis miles*) and *Gaeolaelaps aculeifer*, as well as the rove beetle *Dalotia coriaria* (formerly *Atheta coriaria*), are commercially produced as biocontrol agents against WFT pupae.^61^, ^62^, ^63^

#### 2.4.4 Parasitoids

To date, *Ceranisus menes* and *C. americensis* are the only two parasitoid wasps investigated for their potential to control WFT.^64^ Under laboratory conditions, these parasitic wasps oviposit into first‐instar larvae, resulting in death of the prepupal stage. However, slow wasp development time hinders efficient WFT control.

#### 2.4.5 Entomopathogens

Entomopathogens used as WFT biocontrol agents consist of nematodes and fungi. The use of various nematode species and strains in the nematode genera *Steinernema* and *Heterorhabditis* against soil‐inhabiting WFT pupae produced low and inconsistent control results.^65^, ^66^ While foliar application of *S. feltiae*, in the presence of a wetting agent, has not been shown to successfully control WFT adults and larvae in chrysanthemum 67, 68, repeated applications successfully reduced thrips damage in cucumber.^69^ Treatment with *Thripinema* nematodes, infecting WFT residing within flower buds and foliar terminals, was non‐lethal and caused sterility of female WFT. This treatment was insufficient for control of WFT.^67^

Entomopathogenic fungal conidia infect thrips by penetrating their cuticle to obtain nutrients for growth and reproduction. In general, adult thrips are more susceptible than larval and pupal stages, possibly because moulting avoids contact with fungal inoculum. In addition, larvae have thicker cuticles, which may delay penetration of fungus. Foliar applications of different fungal strains belonging to *Beauveria bassiana*, *Metarhizium anisopliae* and *Lecanicillium lecanii* (formerly *Verticillium*) significantly reduced thrips populations in greenhouse vegetable and floral crops.^70^, ^71^ Besides the direct effects, *B. bassiana* showed sublethal effects on the progeny of treated WFT adults.^72^ Several formulations of entomopathogenic fungi are now available for foliar applications, but their efficacy has been inconsistent, likely owing to varying ambient humidity and temperature. Formulations targeting the soil stage have shown promising results in potted chrysanthemum.^73^ Major constraints to the use of entomopathogenic fungi as augmentative biological control agents remain the difficulties in mass production, storage and formulation.^74^ Recently, the use of endophytic fungi, developing within plant tissues without causing disease symptoms, has been explored for WFT control. So far, no negative effects on WFT preference or development have been observed.^75^, ^76^

#### 2.4.6 Combinatorial use of biological control

Combinatorial treatments of natural enemies with different arthropods or arthropods with entomopathogens are used as alternative or back‐up treatments. This requires careful timing and compatibility of treatments. Application of *A. swirskii* together with *N. cucumeris* in laboratory trials led to negative interactions on WFT control through intraguild predation.^77^ Simultaneous use of predatory mites and pirate bugs did have a negative effect on WFT in greenhouse crops, but the effect was no greater than using one predator alone.^58^, ^78^ In contrast, a combination of *O. laevigatus* and *Macrolophus pygmaeus*, a generalist predator to control aphids, achieved enhanced control of both thrips and aphids in sweet pepper.^79^ Combinations of the entomopathogenic fungus *B. bassiana* with predatory mites did not inhibit or enhance the control of WFT, because fungal dissemination seemed to be hindered by mite grooming.^70^, ^80^

Thrips generally complete their life cycle within 2 weeks, causing several generations to overlap during a single crop production cycle. Hence, combinations of foliar and soil‐dwelling biocontrol agents targeting all WFT life stages have been investigated. Simultaneous treatment of different mites or pirate bugs as foliage predators with the soil predators *G. aculeifer*, *D. coriaria* or the nematode S. *feltiae* did not reduce thrips numbers in ornamentals beyond that caused by foliage predators alone.^81^ In contrast, the use of *Heterorhabditis* nematodes with the foliar‐dwelling mite *N. cucumeris* provided superior control in green bean compared with individual releases.^82^ Combinations of different predatory mites with the nematode *S. feltiae* achieved good WFT control in cyclamen, while combinations of *O. laevigatus* with the respective nematodes failed to control thrips.^59^ Likewise, laboratory combinations of different soil‐dwelling predators with *S. feltiae* did not improve thrips control, while combinations of these predators with the entomopathogenic fungi *M. brunneum* and *B. bassiana* achieved higher control of WFT compared with single treatments.^83^ Concurrent use of the soil‐dwelling mite *H. aculeifer* with the nematode *S. feltiae* increased mortality of WFT pupae in green bean.^84^ It is apparent that combinations of biocontrol agents for control of WFT are promising but require careful management and fine‐tuning suiting the crop in question.

### 2.5 Behavioural control

An important focus in applied pest control is the manipulation of adult insect behaviour using semiochemicals functioning as signal compounds. Pheromones serve for intraspecific communication between arthropods, while allelochemicals mediate plant–insect interactions. Semiochemicals are used as lures for monitoring as well as for control purposes.

#### 2.5.1 Pheromones

Two key pheromones in male WFT were identified: (*R*)‐lavandulyl acetate and neryl (*S*)‐2‐methylbutanoate.^85^ The latter is a sexual aggregation pheromone attracting both male and female WFT. The synthetic analogues Thripline AMS (Syngenta Bioline, Clacton, UK) and ThriPher (Biobest, Westerlo, Belgium) are in use commercially. Decyl and dodecyl acetate, 10‐ and 12‐AC respectively, are produced as alarm pheromones in anal larval droplets. Synthetic equivalents caused WFT to increase movement and take‐off rates, reduce oviposition and reduce landing rates, suggesting their function as an alarm pheromone.^86^, ^87^ More recently, 7‐methyltricosane, a WFT‐male‐specific cuticular hydrocarbon, was suggested to inhibit mating.^88^

#### 2.5.2 Allelochemicals

Volatiles used to locate plant hosts for feeding and oviposition can be applied as lures. Various volatile scents, including benzenoids, monoterpenes, phenylpropanoids, pyridines and a sesquiterpene, attracted adult female *F. occidentalis* in a dose‐dependent way.^89^ While WFT were attracted by pure linalool as well as linalool emitted by engineered chrysanthemum plants, they were deterred by linalool glycosides.^89^ The latter may represent a plant defence strategy against WFT as a floral antagonist, balancing attractive fragrance with poor taste. Methyl isonicotinate, the active ingredient of Lurem‐TR (Koppert Biological Systems, Berkel en Rodenrijs, The Netherlands), is an attractant for both male and female WFT as well as other thrips species and is used to locate host plants.^91^ Recently, a new potential active ingredient for thrips lures, volatile (*S*)‐verbenone, was described from pine pollen.^92^ Volatiles with repellent activities can be utilised for disruption of host finding. Applications of methyl‐jasmonate and cis‐jasmone deterred WFT larvae from feeding and settling, although repeated exposure resulted in a dose‐dependent habituation.^93^, ^94^ The monoterpenoid phenols thymol and carvacrol exhibited both a feeding as well as a oviposition deterrent effect to WFT.^95^, ^96^

Currently, the three commercially available WFT semiochemicals are mainly used as lures in conjunction with sticky card traps. Adult thrips constantly explore their host range for feeding and reproduction by utilising different cues, including volatiles. Therefore, semiochemicals hold great promise for thrips mass trapping as well as ‘lure and kill’ strategies.^97^, ^98^ Combination of dodecyl acetate with maldison, an organophosphorous insecticide, increased larval mortality of WFT.^99^ Use of LUREM‐T together with the WFT predator *O. laevigatus* increased the abundance of the latter.^100^ The ‘lure and infect’ strategy employs LUREM‐T for autodissemination of the entomopathogenic fungus *M. anisopliae* by attracting thrips to particular traps provided with fungal inoculum.^101^

### 2.6 Chemical control

Chemical control is among one of the most frequently used methods to suppress WFT, particularly for ornamentals, where an almost zero damage tolerance encourages intensive application of insecticides. Commonly used insecticides for management of thrips, approved at European level, are listed in Table 2.

**Table 2:** Overview of synthetic and natural compounds used against thrips, based on commercial spray advice cards 2015

		Type of compound	Trade name	Target	Crops
Natural origin		Pyrethrins	Spruzit/Raptol	Sodium channel	Lettuce, cutflowers, strawberry
		Azadirachtin	NeemAzal	Ecdysone receptor	Rose, chrysanthemum, cutflowers
Synthetic origin	Selective chemicals	Pyridalyl	Nocturn	Protein synthesis	Rose
		Lufenuron	Match	Chitin biosynthesis	Rose, cutflowers
	Broad chemical spectrum	Spinosad	Conserve	Nicotinic acetylcholine receptor	Capsicum, rose, cutflowers, lettuce, cucumber, strawberry
		Abamectin (avermectin, milbemycin)	Vertimec	Glutamate‐gated chloride channel	Capsicum, chrysanthemum, rose, cutflowers, lettuce, strawberry
		Thiametoxam	Actara	Nicotinic acetylcholine receptor	Chrysanthemum, rose, cutflowers
		Methiocarb	Mesurol	Acetylcholinesterase	Chrysanthemum, rose, cutflowers
		Esfenvaleraat	Sumicidin	Sodium channel	Chrysanthemum, rose, cutflowers
		Deltamethrin	Decis EC	Sodium channel	Capsicum, chrysanthemum, rose, cutflowers, lettuce, cucumber, strawberry
		Spirotetramat	Movento	Acetyl CoA carboxylase	Chrysanthemum

Management of thrips has relied on the application of insecticides, as has been described in previous reviews, to which we refer for further detail.^4^, ^7^ The use of broad‐spectrum insecticides, including pyrethroids, neonicitinoids, organophosphates and carbamates, kills native outcompeting thrips species and natural enemies disrupting WFT management.^1^, ^4^, ^5^, ^6^, ^7^, ^102^ Spinosad, a natural reduced‐risk insecticide derived from an actinomycete bacterium, is compatible with natural enemies and currently provides the most effective chemical control of WFT.^4^ New, narrow‐spectrum insecticides for WFT control include pyridalyl and lufenuron. However, frequent applications of broad‐ and narrow‐spectrum insecticides, including spinosad, have led to the development of WFT resistance to active ingredients of most chemical classes, as has been extensively revised elsewhere.^5^, ^6^, ^103^ Management of WFT insecticide resistance as reviewed in other publications comprises resistance monitoring coupled with rotations among different classes of insecticides.^5^, ^6^ However, development of rotation schemes does not necessarily focus on reducing overall insecticide use. Therefore, insecticides should only be used if economic damage thresholds are reached, and here applications should be accurate and precise while conserving natural enemies. Rotation schemes need to be complemented with other compatible control approaches.^5^ Rotation programmes including entomopathogenic organisms successfully controlled WFT under greenhouse conditions.^104^ Various insecticides have been shown to be compatible with WFT predatory mites, bugs and other competing thrips species.^104^, ^105^

## 3 FUTURE DIRECTIONS OF WFT CONTROL: ‘OMICS’ TECHNOLOGIES

In pest management programmes, innovative approaches advancing the prevention and management of pest insects are constantly being sought. The development of non‐targeted analytical methods, from genomes to metabolites, has been a major driver for the adaptation of systems‐based approaches. Such integrative approaches enable a comprehensive view of defence mechanisms. The emergence of ‘omic’‐based techniques, as well as advances in computational systems, provides a powerful tool to drive innovation in crop protection. Understanding plant–insect interactions, genetic variations among insect populations and resistant crop varieties generates valuable information that provides new opportunities and technologies by improving our knowledge of complex resistance traits.

### 3.1 Plant genomics

While domestication of wild plants through selection improved yield and palatability, it greatly reduced phenotypic and genetic diversity, leading to loss of insect resistance. Wild ancestors therefore provide a promising source for breeding of WFT resistance traits.^32^, ^35^ Moreover, the presence of considerable variation in resistance to WFT between accessions, as observed in various vegetables and ornamentals, can be exploited as well.^32^, ^35^, ^36^, ^106^ Identifying sets of genes or metabolites as biomarkers enables the introduction of novel insect resistance traits into breeding lines. In a highly resistant pepper accession, a quantitative trait locus (QTL), mapped to chromosome 6, confers resistance to WFT by affecting the larval development of thrips.^107^ This approach, however, might be less suitable for polyploid ornamentals. At present, successful breeding of resistant cultivars is limited to TSWV control. Genes known to confer resistance against TSWV isolates include *Sw‐5* (*L. peruvianum*), *Sw‐7* (*L. chilense*) and *Tsw* (*C. chinense*).^108^, ^109^

### 3.2 Insect genomics

Despite their economic importance as worldwide crop pests, the ‘i5k’ (5000 insect genome) project has only recently developed genomic and proteomic tools for WFT, including a collection of assembled and annotated sequences.^110^, ^111^ The availability of the thrips genome will open up new powerful opportunities to elucidate thrips gene function and develop alternative control strategies based on the molecular interaction of thrips with plants as well as viruses.^112^ An RNA interference tool has been developed using microinjection for delivery of double‐stranded RNA into adult thrips.^113^ Targeting the vacuolar ATP synthase subunit‐B gene resulted in increased WFT mortality and reduced fecundity of surviving females. Alternatively, symbiont‐mediated RNAi, downregulating an essential tubulin gene, resulted in high mortality of WFT larvae.^114^ For transmission of TSWV, a suite of WFT candidate proteins reacting to viral infection has been identified, but no RNAi approach for disruption has yet been developed.^110^ Sequencing the salivary gland transcriptome of TSWV‐infected and non‐infected WFT led to the putative annotation of genes involved in detoxification and inhibition of plant defence responses.^111^ The availability of WFT genome and transcriptome sequence data will facilitate the development of approaches identifying thrips effectors suppressing or inducing plant defence responses.

### 3.3 Metabolomics

Metabolomics has a great potential to detect a wide range of compounds in an unbiased or untargeted fashion. So far, metabolomics has mainly been restricted to comparative approaches using genotypes with contrasting levels of resistance, classified as resistant or susceptible.^34^ Addressing the metabolome, however, allows investigation of the complex and integrated network underlying defence mechanisms. Combined with genetic approaches, metabolomics analyses provide powerful opportunities to identify metabolic markers for resistance to thrips and open up possibilities of ‘metabolite breeding’. Identification of compounds conferring resistance to different herbivores, i.e. cross‐resistance, could form a basis for a multiresistance breeding programme. An overlap of resistance to WFT and celery leafminer (*Liriomyza trifolii*) has been described in chrysanthemum.^106^ Manipulation of environmental factors may increase concentrations of resistance‐related metabolites within plants, thereby enhancing WFT control. Rutin and chlorogenic acid, two phenolic compounds involved in thrips resistance, are enhanced upon UV‐B exposure.^115^ In addition, plant secondary metabolites involved in WFT resistance could be used to develop new protection agents that enhance or activate the plants' own defence mechanisms or that may provide new mode of actions with improved selectivity, minimising the effects on non‐target organisms.

Next to plants, microbials offer a huge source of metabolites to be used for insect resistance. Assembly of microbial communities may influence the performance of thrips through plant chemistry or volatile emission. Colonisation of onion seedlings by fungal endophytes induced resistance to *Thrips tabaci*, likely owing to a repellent effect of volatiles.^116^ Investigations into endophytes increasing resistance to WFT have not been successful so far.^75^, ^76^ Rhizobacteria are known to play an important role in plant growth, nutrition and health in general. Genetic variation in response to the capacity of plants in reacting to these beneficial bacteria opens the way for breeding of plants maximising bacterial benefits. The effect of soil microbial communities on plant above‐ground defence directed against insects, such as thrips, still needs to be explored. Similarly, the effect of the bacterium *Pseudomonas syringae* producing the JA analogue coronatine and thus triggering herbivore defence has a potential to be explored for plant defence to WFT.^117^

### 3.4 High‐throughput screening

Employing genomic as well as metabolomics techniques, however, requires a high‐throughput screening (HTS) system for thrips resistance. Screening large numbers of plants for identification of resistance sources is vital for resistance breeding programmes.^118^ Recently, a high‐throughput phenotyping method has been described that uses automated video tracking of WFT behaviour.^119^ However, a reproducible high‐throughput method assessing thrips damage is still lacking. Similarly, HTS systems testing for active metabolites against WFT deriving from plants or microbials are absent. Development of stable thrips‐derived cell lines, beyond primary cell cultures, has been unsuccessful until now.^120^ However, the availability of the thrips genome sequence provides an unprecedented opportunity to identify gustatory or olfactory receptors to form the basis of HTS development.

## 4 CONCLUSIONS

As from 2014, farmers in the EU are obliged to implement the principles of integrated pest management. However, despite the various benefits expected from IPM, there seems to be little evidence that IPM has been largely adopted. Many studies seek to develop their respective methods as single‐solution approaches to pest problems rather than integrating these into an ‘IPM toolbox’. Moreover, vertical integration of control measures looking at IPM of different pests in one cropping system is scarce.^7^ Developing and implementing IPM remains a complex knowledge‐based task. Integrating different control tactics is fundamental to achieving successful control of WFT, yet it presents significant challenges. Clearly, research into the integration of methods involves cooperative, jointly planned activities that cannot be pinned down into a single methodological blueprint. How can scientists in different groups develop protocols and tests that allow the combination of multiple approaches in sustainable pest management while retaining the capacity to determine the individual contributions and hence modify and improve these? For optimal effectiveness and progress, strategies should not only be integrated at inter‐ and multidisciplinary research levels but also driven through applied outcomes in cooperation with commercial partners by transdisciplinary research.

Significant research progress in control of WFT has been made. Host plant resistance to WFT becomes increasingly important. Some breeders already have varieties with different resistance ratings; however, for certain crops, such as polyploid ornamentals, this approach is not as straightforward. Recently, more emphasis has been placed upon biological control of WFT in protected crops. Nevertheless, short crop cycles and low thresholds, for ornamentals in particular, make biological control challenging. Another promising approach is the use of semiochemicals, not only for monitoring but also for thrips control. Looking to the future, there are many exciting (bio)technological advances that will undoubtedly boost the control of thrips. With the ‘omics’ revolution, we have the tools at hand to fully grasp this potential. Nevertheless, much remains to be learned about plant–insect interactions to make further important contributions to the development of environmentally friendly, biologically sustainable crop protection strategies against thrips. Molecular modifications, genetic engineering and the development of novel biological products, including microorganisms and metabolites, will allow the development of improved cultivars that are able to respond to WFT attack by enhancing resistance. However, not only should new strategies be explored, but existing ones should be viewed in the context of IPM programmes, with the emphasis on compatibility as well as on ecological, environmental and economic consequences. Looking at different crops, it becomes even more complex. In crop protection, as in life, one size does not fit all. In order to achieve successful control, strategies should be tailored to fit the requirements of different production systems. Controlling pests is not a trivial issue, and has never been. The basic question remains of how one achieves consistent long‐term control. Most importantly, there remains the need for transdisciplinary approaches integrating different practices for control of thrips.
